# New strategies with anti-IgE in allergic diseases

**DOI:** 10.1186/1939-4551-7-17

**Published:** 2014-07-29

**Authors:** Stephen T Holgate

**Affiliations:** 1 Clinical and Experimental Sciences, Faculty of Medicine, University of Southampton, Southampton General Hospital, Mail point 810, Level F, South Block, Southampton SO166YD, UK

**Keywords:** Omalizumab, Anti-IgE monoclonal antibody, Asthma, Comorbidity, Allergy

## Abstract

IgE has long been known as a therapeutic target for allergic disease, but the difficulty has been in selecting agents that don't trigger cross linkage of IgE when bound to its high affinity receptor (FceR1) on mast cells and basophils. By “designing” a monoclonal antibody (mAb) which targets that part of IgE that binds to that binds to the a-chain of FceR1, the allergic cascade can be effectively interrupted and diseases such as asthma greatly improved, providing a substantial part of their phenotype engages IgE. Clinical trials and real life studies confirm this. Beyond asthma, a whole range of other diseases dependent upon IgE initiation and triggering are being identified. These diseases are now being explored as being amenable to anti-IgE therapy some of which are comorbidities of asthma and others not. The advent of an even more potent anti-IgE mAb - QGE031 – is creating further opportunities for anti-IgE therapy to improve the lives of so many people with IgE-related diseases.

## Introduction

### IgE as a therapeutic target in allergic disease

Carl Prausnitz and Heinz Küstner first described the passive transfer of the allergen-specific response in the skin with serum in 1921 [1]. They called the “substance” present in the serum *reagin*. It took a further 45 years before Kimishige Ishizaka, Gunnar Johansson and Hans Bennich identified *reagin* as the 5^th^ immunoglobulin class, immunoglobulin (Ig)E [2]. Subsequently, the mechanism of cell activation through which cross-linkage of cell bound IgE on mast cells and basophils was shown to involve dimerisation of the high affinity IgE receptor, FcϵR1, leading to non-cytotoxic degranulation and generation of newly formed lipid mediators responsible for the acute allergic response. The later recognition that both mast cells and basophils can also release preformed and newly generated cytokines, chemokines and growth factors helps explain how the acute allergic response transits into late-phase and more chronic responses associated with leukocyte recruitment and activation of tissue remodelling pathways [3]. More recently FcϵR1 has also been found on antigen-presenting dendritic cells (DCs) where they function to facilitate the uptake and processing of allergens to enhance sensitisation [4]. Thus, identification of IgE as a therapeutic target in allergic diseases such as asthma has been known for many years, but the difficulty has been in identifying therapeutic agents that could block its effects.

### Anti-IgE mAbs; the first biologics for treating allergic diseases

A major breakthrough occurred when it was shown that the Cϵ3 region of the Fc fragment of IgE binds very selectively to a particular component of the α-chain of the tetrameric FcϵR1 (α_1_β_1_γ_2_) [5]. This enabled blocking immunoglobulin to be raised against IgE and specifically to the Cϵ3 region and as a consequence avoid cross-linking of IgE bound to its high affinity receptor. Starting in mice [6], a chimeric IgG mAb active in humans was produced and shown to be effective in reducing circulating IgE without causing an anaphylactic response [7]. By humanising IgG1 anti-human IgE mAb containing only the antigen binding site as mouse sequences, omalizumab was born [8]. After successful Phase 2 proof of concept studies on allergen induced EAR and LAR and allergen-triggered skin wheal and flare reaction, omalizumab successfully entered the clinic for the treatment of severe allergic asthma in both adults and children over the age of 12 years reviewed in [9].

### Omalizumab clinical profile

With a good safety record, many clinical studies confirmed efficacy in severe asthma which have now extended to “real world” studies [10]. Lingering concerns about omalizumab and increased malignancy have recently been largely dispelled by the large postmarketing EXCELS (Evaluating Clinical Effectiveness and Long-term Safety in Patients with Moderate-to-Severe Asthma) study indicating that omalizumab therapy is not associated with an increased risk of malignancy [11]. As with other humanised antibodies, there remains, however occasional anaphylactic-type responses which sometimes can be delayed for which a mechanism has yet to be found [12].

**Figure 1:**
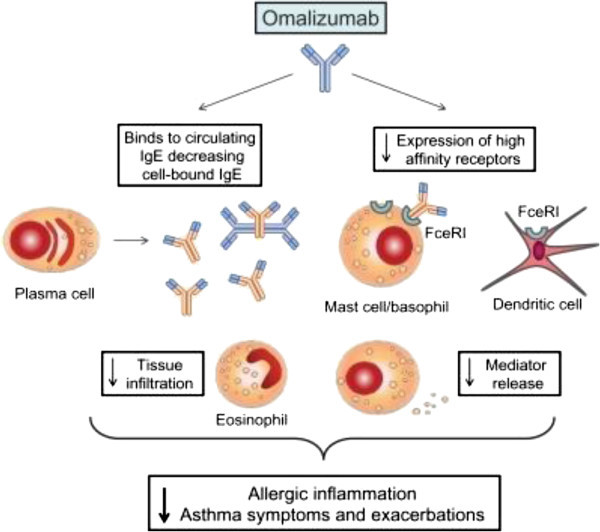
Reduce levels of circulating free IgE by interacting with omalizumab to form immune complexes prevents IgE interaction with cell surface high-affinity IgE receptors (FcóϵRI) expressed on dendritic cells, mast cells and basophils and also results in inhibition of FcϵRI expression to enhance inhibitory effects on the allergic cascade.

Two issues are important to recognise in the clinical use of omalizumab; the dosing according to the circulating levels of serum total IgE (700 iu/ml being the upper limit for inclusion) and body weight and the responder/non-responder patients that so far cannot be differentiated on the basis of known biomarkers. The latter problem mandates an assessment of efficacy after 16 weeks of treatment using both practitioner and patient assessment of outcome measures [13]. Another important feature of omalizumab’s clinical efficacy is the formation of IgG/IgE trimeric and hexameric immune complexes without complement activation or any other toxicity. High levels of circulating IgE results in an increase in FcϵR1 on effector cells whereas removal of IgE with omalizumab causes down-regulation of FcϵR1 on these cells and undoubtedly contributes to the efficacy of this treatment [14]. Blockade of IgE reduces the allergic inflammatory cascade including eosinophil and basophil recruitment probably by combined effects influencing allergen sensing and processing by airway DCs (Figure 1) [15] and blockade of mediator secreting cells. However, counter intuitively, inhibition of these cellular and mediator events with omalizumab does not appear to reduce non-specific airways responsiveness (such as methacholine and histamine PC_20_) at least in the intermediate term [16], but do inhibit measures of indirect responsiveness such as adenosine and exercise which involve mast cell activation [17]. This might suggest that internalisation of FcϵR1induced when IgE is removed results in “quietening” of mast cell releasability to stimuli other than mediated directly through IgE e.g. the adenosine A_2b_-receptor mast cell activation pathway.

A fascinating aspect of the therapeutic response of omalizumab in severe allergic asthma is the powerful effect it has on asthma exacerbations, patient-related and quality of life measures, but less effect on measures of lung function. Part of the reason for this relatively poor response on lung function could be the result of prior maximum bronchodilator and anti-inflammatory effects of corticosteroids and long-acting β_2_-agonists (LABAs) which all patients being selected for treatment with omalizumab will be receiving. However, what is most gratifying is the ability in both adults and children for oral and high dose inhaled corticosteroids to be reduced and, in some cases, stopped altogether with a reduction in corticosteroid comorbidities without loss of asthma control [18,19]. Also of importance is the marked effect of omalizumab in reducing emergency physician consultations, courses of oral corticosteroids and hospital admissions associated with severe exacerbations.

Although severe asthma in children is less frequent than in adults, when it does occur it is most frequently associated with atopy and atopic comorbidities such as atopic dermatitis, allergic rhinitis and food allergy. Omalizumab is equally effective in children [20]. Omalizumab, therapy also reduces the September epidemic of childhood asthma in inner city US severe asthma thereby strengthening link between allergic and viral mechanisms in such children [21].

### Does omalizumab influence airway remodelling in chronic asthma?

A key question is what the long-term effect of omalizumab is on airway wall remodelling in chronic asthma. This has been approached by assessing airway wall thickness by computed tomography (CT). One study by Hoshino and colleagues [22] conducted on 30 patients with severe persistent asthma showed that omalizumab for 16 weeks significantly reduced airway wall area that correlated with reduced sputum eosinophila and increase in baseline lung function. A further study has shown that 50% of omalizumab responders exhibit a reduction in epithelial reticular basement membrane (RBM) thickening suggesting effects on remodelling [23].

### Systemic actions of omalizumab on asthma comorbidities and allied “allergic” conditions

**Figure 2:**
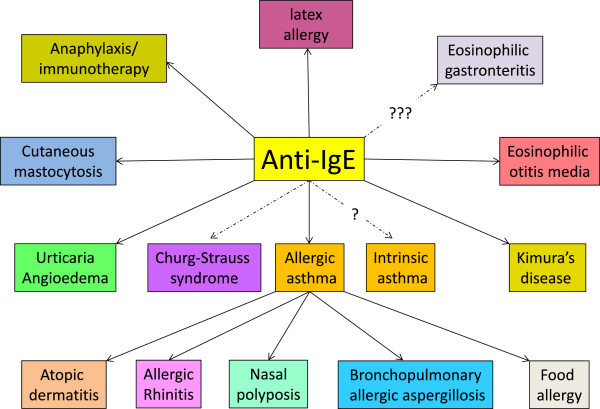
**Diseases in which anti-IgE therapy with omalizumab has been claimed to be efficacious.** Intrinsic (or non-allergic asthma) has revealed efficacy in some anecdotal reports.

In administering an anti-allergy therapy systemically, there are likely to be powerful effects on other allergic manifestations in addition to asthma (Figure 2) [24]. Some randomised and observational trials, as well as case studies, have shown that omalizumab is efficacious in patients with allergic rhinitis, bronchopulmonary allergic aspergillosis, eosinophilic otitis media, urticaria, Kimura’s disease (subcutaneous granuloma of soft tissues in the head and neck region, increased eosinophil counts and high serum IgE levels), food allergy and idiopathic and exercise-induced anaphylaxis, atopic dermatitis, protection from anaphylaxis during allergen immunotherapy, latex allergy, cutaneous mastocytosis, eosinophilic gastroenteritis, nasal polyposis and even some cases of intrinsic asthma (Figure 2, Table 1). Of these, the dramatic effect of anti-IgE treatment of chronic urticarial and angioedema is especially valuable since the therapeutic options for such patients are very limited beyond anti-histamines, oral corticosteroids and immunosuppressants [25]. While urticarial can occur alongside asthma when it is associated with atopy, especially food allergy, it most frequently occurs as an “idiopathic form” where no known external allergies are found; nevertheless anti-IgE therapy can still be highly effective in such patients event in the absence of autoantibodies against IgE or FcϵR1 (see Chapter by Sarbjit Saini in this series of reviews). While all of these indications must indicate some role for IgE in pathophysiology, this may not always be obvious (as in urticaria). One possibility is that the quieting of mast cell releasability with loss of the cell surface FcϵR1 during omalizumab treatment raises the threshold for mast cell activation independent of the stimulus. Such beneficial effects across a range of allergic disorders is likely to be an important factor in the improvement in quality of life and wellbeing encountered in the severe allergic asthma trials and mandates the derivation of a new set of outcome measures that capture these in complex multisystem allergy as has proven so useful in other complex immunological diseases such as rheumatoid arthritis and SLE.

**Table 1:** Diseases in which ant-IgE has been reported to exhibit efficacy

**Disease**	**Responsiveness to omalizumab**	**Reference**
Non-allergic asthma	Some reported benefits, but controversial	- Menzella F, Piro R, Facciolongo N, Castagnetti C, Simonazzi A, Zucchi L. Long-term benefits of omalizumab in a patient with severe non-allergic asthma. Allergy Asthma Clin Immunol. 2011; 7: 9.
		- Domingo C, Pomares X, Angril N, Rudi N, Amengual MJ, Mirapeix RM. Effectiveness of omalizumab in non-allergic severe asthma. J Biol Regul Homeost Agents. 2013; 27: 45–53.
Churg-Strauss (C-S) Syndrome	Anecdotal evidence of efficacy, but also reports of uncovering latent C-S disease	- Giavina-Bianchi P, Giavina-Bianchi M, Agondi R, Kalil J.Administration of anti-IgE to a Churg-Strauss syndrome patient. Int Arch Allergy Immunol. 2007; 144: 155–8.
		- Wechsler ME, Wong DA, Miller MK, Lawrence-Miyasaki L. Churg-strauss syndrome in patients treated with omalizumab. Chest. 2009; 136: 507–18.
Allergic rhinitis	Well documented benefit, but questionable cost-effectiveness	- Vashisht P, Casale T. Omalizumab for treatment of allergic rhinitis. Expert Opin Biol Ther. 2013; 13: 933–45.
Atopic dermatitis (AD, eczema)	Efficacious in severe refractory AD	- Kim DH, Park KY, Kim BJ, Kim MN, Mun SK. Anti-immunoglobulin E in the treatment of refractory atopic dermatitis. Clin Exp Dermatol. 2013; 38: 496–500.
		- Sánchez-Ramón S, Eguíluz-Gracia I, Rodríguez-Mazariego ME, Paravisini A, Zubeldia-Ortuño JM, Gil-Herrera J, Fernández-Cruz E, Suárez-Fernández R. Sequential combined therapy with omalizumab and rituximab: a new approach to severe atopic dermatitis. J Investig Allergol Clin Immunol. 2013; 23: 190–6.
Nasal polyposis	Efficacious in allergic and non-allergic polyposis	- Gevaert P, Calus L, Van Zele T, Blomme K, De Ruyck N, Bauters W, Hellings P, Brusselle G, De Bacquer D, van Cauwenberge P, Bachert C. Omalizumab is effective in allergic and nonallergic patients with nasal polyps and asthma. J Allergy Clin Immunol. 2013; 131: 110–6.
Bronchopulmonary allergic aspergillosis	Limited evidence. Requires further study	- Jat KR, Walia DK, Khairwa A. Anti-IgE therapy for allergic bronchopulmonary aspergillosis in people with cystic fibrosis. Cochrane Database Syst Rev. 2013; 9: CD010288.
Food allergy	In conjunction with allergen immunotherapy	- Schneider LC, Rachid R, LeBovidge J, Blood E, Mittal M, Umetsu DT. A pilot study of omalizumab to facilitate rapid oral desensitization in high-risk peanut-allergic patients. Allergy Clin Immunol. 2013; 132: 1368–74.
		- Nadeau KC, Schneider LC, Hoyte L, Borras I, Umetsu DT. Rapid oral desensitization in combination with omalizumab therapy in patients with cow's milk allergy. J Allergy Clin Immunol. 2011; 127: 1622–4.
Chronic (idiopathic) urticaria and angioedema	Active independent of known engagement of IgE-driven pathways	- Maurer M, Rosén K, Hsieh HJ, Saini S, Grattan C, Gimenéz-Arnau A, Agarwal S, Doyle R, Canvin J, Kaplan A, Casale T. Omalizumab for the treatment of chronic idiopathic or spontaneous urticaria. N Engl J Med. 2013; 368: 924–35.
		- Lang DM. A critical appraisal of omalizumab as a therapeutic option for chronic refractory urticaria/angioedema. Ann Allergy Asthma Immunol. 2014; 112: 276–9.
Kimura’s disease	Anecdotal evidence	- Nonaka M, Sakitani E, Yoshihara T. Anti-IgE therapy to Kimura's disease: A pilot study. Auris Nasus Larynx. 2014; 41: 384–8.
Eosinophilic otitis media	Limited evidence for efficacy	- Iino Y, Hara M, Hasegawa M, Matsuzawa S, Shinnabe A, Kanazawa H, Yoshida N. Clinical efficacy of anti-IgE therapy for eosinophilic otitis media. Otol Neurotol. 2012; 33: 1218–24.
		- Iino Y, Hara M, Hasegawa M, Matsuzawa S, Shinnabe A, Kanazawa H, Yoshida N. Effect of omalizumab on biomarkers in middle ear effusion in patients with eosinophilic otitis media. Acta Otolaryngol. 2014; 134: 366–72.
Mastocytosis	Anecdotal case reports	- Kibsgaard L, Skjold T, Deleuran M, Vestergaard C. Omalizumab Induced Remission of Idiopathic Anaphylaxis in a Patient Suffering from Indolent Systemic Mastocytosis. Acta Derm Venereol. 2014; 94: 363–364.
		- Matito A, Blázquez-Goñi C, Morgado JM, Alvarez-Twose I, Mollejo M, Sánchez-Muñoz L, Escribano L.Short-term omalizumab treatment in an adolescent with cutaneous mastocytosis. Ann Allergy Asthma Immunol. 2013; 111: 425–6.
Eosinophilic gastroenteritis and oesophagitis	Probably ineffective	- Rocha R, Vitor AB, Trindade E, Lima R, Tavares M, Lopes J, Dias JA. Omalizumab in the treatment of eosinophilic esophagitis and food allergy. Eur J Pediatr. 2011; 170: 1471–4.
		- Stone KD, Prussin C. Immunomodulatory therapy of eosinophil-associated gastrointestinal diseases. Clin Exp Allergy. 2008; 38: 1858–65.
Latex allergy	Limited evidence for efficacy	- Leynadier F, Doudou O, Gaouar H, Le Gros V, Bourdeix I, Guyomarch-Cocco L, Trunet P. Effect of omalizumab in health care workers with occupational latex allergy. J Allergy Clin Immunol. 2004; 113: 360–1.
Anaphylaxis (including idiopathic and exercise-induced)	Anecdotal.	- Bray SM, Fajt ML, Petrov AA. Successful treatment of exercise-induced anaphylaxis with omalizumab. Ann Allergy Asthma Immunol. 2012; 109: 281–2.
	Also in conjunction with allergen-specific immunotherapy	- Demirtürk M, Gelincik A, Colakoğlu B, Dal M, Büyüköztürk S. Promising option in the prevention of idiopathic anaphylaxis: omalizumab. J Dermatol. 2012; 39(6): 552–4.

### A new more potent anti-IgE mAb in development

An exciting new development is the production of a much more potent form of anti-IgE, QGE031 (Novartis), which is at least 12 times more potent than omalizumab for the treatment of IgE-driven diseases where a significant unmet need exists such as severe uncontrolled asthma, atopic dermatitis and food allergies [26]. Bullous pemphigoid is also a potential disease with high morbidity in which QGE031 could be active on account of its increased activity on IgE-mediated skin responses and its prolonged duration of action [27].

In conclusion:

– Clinical trials have repeatedly shown that anti-IgE is highly effective in reducing exacerbations and reducing corticosteroid use, as well as baseline control of asthma in adults and children.

– Reduced use of health services such as hospital admission, ICU etc. makes the economic case for omalizumab in managing high risk severe asthma.

– Omalizumab is active on asthma comorbidities as well as other conditions, presumably related to allergy and/or IgE.

– There appear to be some long term benefits of omalizumb on airway wall remodelling.

– A more active anti-IgE mAb is in development.

## Competing interests

Stephen Holgate has received honoraria from Novartis for lecturing on anti-IgE treatment and chaired the 2014 Novartis International Respiratory Advisory Council in Amsterdam. He has also contributed to other reviews on anti-IgE therapy.
